# The Effect of Translucency and Surface Treatment on the Flexural Strength of Aged Monolithic Zirconia

**DOI:** 10.1155/2021/8022430

**Published:** 2021-11-09

**Authors:** Rashin Giti, Benika Abbasi

**Affiliations:** Department of Prosthodontics, School of Dentistry, Shiraz University of Medical Sciences, Shiraz, Iran

## Abstract

**Aims:**

This *in vitro* study aimed to evaluate the effect of the degrees of translucency in different types of monolithic zirconia as well as the aging and surface treatment with airborne particle abrasion on the flexural strength of monolithic zirconia.

**Materials and Methods:**

Sixty bar-shaped specimens were fabricated from three different types of presintered monolithic zirconia (*n* = 20 per group) including low translucent (LT) (DD Bio ZW iso, high strength zirconia, Dental Direkt, Germany), high translucent (HT) (DD Bio ZX^2^ 98, high translucent zirconia, Dental Direkt, Germany), and multilayered system (ML) (DD cubeX2®ML, multilayer, cubic zirconia system, Dental Direkt, Germany). Each monolithic zirconia group was equally subdivided according to be either air-abraded with 110 µm aluminium oxide particles or left untreated (control). After thermocycling, the flexural strength was measured by using a universal testing machine. Two-way ANOVA followed by Tukey's post hoc and independent samples *t*-test were used for the statistical analyses (*P* < 0.05).

**Results:**

Surface treatment and types of zirconia were found to have a significant interaction (*P* = 0.010). Having controlled the effect of surface treatment, the flexural strength of HT and LT zirconia was found to be significantly higher than the ML zirconia system (*P* ≤ 0.001). Airborne particle abrasion could significantly decrease the flexural strength of monolithic zirconia only in ML zirconia (*P* = 0.002).

**Conclusions:**

Multilayered zirconia system had the lowest flexural strength among all groups. Moreover, the flexural strength of this system was attenuated by surface treatment with airborne particles abrasion.

## 1. Introduction

The excellent mechanical properties of zirconia have promoted it to a great substructure for fixed dental prosthesis, long-span restorations, and implant abutment [1, 2]. Its strength is affected by different stresses such as sintering temperatures, occlusal adjustment, mechanical forces, and surface treatments like grinding or airborne particle abrasion (APA) [2, 3]. Pure zirconia exists in three stable crystallographic forms defined as monolithic (≤ 1170°C), tetragonal (1170 to 2370°C), and cubic phase (2370°C up to melting point). Phase change (tetragonal to monolithic) is capable of transformation toughening due to thermal stress or surface treatments [2, 3]. This phenomenon is accompanied by the reorganization in the lattice at a temperature above 1170°C (1). Transformation toughening is of great importance as it highly contributes to a desirable marginal fit [4]. Among the substances with a stable tetragonal phase at room temperature (CaO, MgO, Y_2_O_3_), yttrium oxide (Y_2_O_3_) is the most common stabilizing oxide, which is added to pure zirconia at a rate of 2–5 molar percentage [2, 3].

Bilayered core-ceramic systems were designed to cover the opaque face of zirconia under a tooth-like appearance while having the strength of yttria-stabilized tetragonal zirconia polycrystalline (Y-TZP). A common complication of bilayered zirconia is porcelain veneer chipping due to the dense and unreactive surface of zirconia and its lower adhesion to ceramic veneers [2, 4, 5], as well as cohesion failure and residual stresses due to mismatch of thermal expansion coefficient and flexural strength between the veneer and Y-TZP core [4–6]. Nonetheless, monolithic zirconia has the advantages of less teeth preparation and fabrication time, material thickness, high strength, and toughness in solid-sintered zirconia, as well as eliminating the complications of porcelain veneer sintering. These prostheses are made with computer-aided design (CAD) and computer-aided manufacturing (CAM) technology [7, 8].

The inherent semitranslucency of monolithic zirconia impedes a natural tooth-like appearance [9]; however, improvements of zirconia block have paved the way towards multilayered shades [8]. Since alterations in elements affect the zirconia properties [10], the optical properties of monolithic zirconia have been enhanced by higher amounts of cubic zirconia and 6 to 8% of yttrium. The translucency of dental ceramics is determined by scattering lightweight, that is, highly scattering ceramics are more opaque. This rate of transmission, absorption, or reflection is expounded to the ceramic microstructure [9, 11]. Core translucency is an esthetically crucial factor when choosing materials [12].

Attaining information regarding the flexural strength is of importance, particularly the mechanical strength of monolithic zirconia after surface treatments [7, 13]. Long-term fatigue results in the propagation of the microcracks caused by airborne particle abrasion [14]. Although the impact of all grinding procedures on the flexural strength was reported to be negligible [15], some research reported that removing the surface or subsurface defects by grinding or polishing increased the dental ceramics strength [16–19]. Airborne particle abrasion was reported to significantly increase the flexural strength due to the ability of zirconia transformation on the surface [15, 20]. APA triggers the monoclinic phase on the zirconia surface, and the resultant surface roughness increases the bond strength with cement materials [9].

Not enough studies have addressed the effect of different translucencies and types of monolithic zirconia especially multilayered system and the combined effect of translucency and surface treatment on the flexural strength of monolithic zirconia. This in vitro study was designed to evaluate the effect of three different translucencies and air abrasion with aluminum oxide (Al_2_O_3_) on the flexural strength of aged monolithic zirconia. The null hypothesis was that different translucencies and surface treatment would not affect the flexural strength of thermocycled monolithic zirconia.

## 2. Materials and Methods

### 2.1. Fabrication of Specimens

In this experimental in vitro study, a bar-shaped specimen (25 × 5 × 2 mm) was designed (CAD design software; 3 shape, Copenhagen, Denmark), based on which 60 specimens were milled (CAD-CAM machine, Cori Tec 340i; imes-icor GmbH, Eiterfeld, Germany) out of three different types of presintered monolithic zirconia (*n* = 20 per group) including low-translucent (LT) monolithic zirconia (DD Bio ZW iso, high strength zirconia, Dental Direkt, Germany), high-translucent (HT) zirconia (DD Bio ZX^2^ 98, high translucent zirconia, Dental Direkt, Germany), and multilayered (ML) system of monolithic zirconia (DD cubeX^2^®ML, multilayer, cubic zirconia system, Dental Direkt, Germany). The specimens were manufactured according to ISO 6872, with ± 0.02 mm accuracy [21] and sintered (ATRA sintering furnace, ATRA Factory, Ghazvin, Iran) following the manufacturer's instruction.

### 2.2. Air-Abrasion

**Figure 1:**
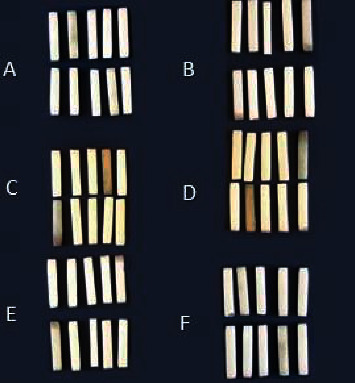
The specimens of three types of monolithic zirconia: (a) hightranslucent zirconia without APA, (b) high-translucent zirconia with APA, (c) low-translucent zirconia without APA, (d) low-translucent zirconia with APA, (e) multilayered zirconia system without APA, and (f) multilayered zirconia system with APA.

Each group was subdivided to be either left untreated as the control or air-abraded with Al_2_O_3_ particles (*n* = 10 per subgroup). Both sides of the specimens were air-abraded with 110 µm laboratory Al_2_O_3_ particles (Renfert Basic Classic, Renfert GmbH, Hilzingen, Germany) for 10 seconds at 400 kPa air pressure from a 10 mm distance. The particle size was adopted with respect to the previous studies claiming that larger particles enhance the surface abrasion, wettability, and cement bond strength [22, 23] (Figure 1).

### 2.3. Scanning Electron Microscopy (SEM)

One specimen of each group was evaluated by scanning electron microscopy (15.0 kV, TESCAN-Vega3 SEM; TESCAN); images were taken at ×500 and ×1500 magnifications to check the surface properties.

### 2.4. Thermocycling and Flexural Strength Test

**Figure 2:**
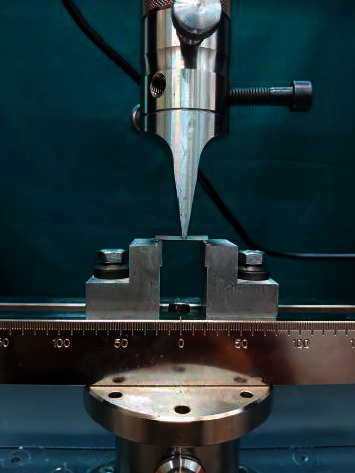
Testing the flexural strength through three-point bend test.

The specimens were, then, subjected to 1000 thermal cycles (Thermocycle, Vafaie Co, Iran) between 5 °C and 55 °C, ultrasonically rinsed with distilled water for 10 minutes, and air-dried for 20 seconds. To measure 3-point flexural strength, a force (Zwick Roell, z020, Germany) was applied on a universal testing machine in the middle of the specimen on a 20 mm fixture at a speed of 1 mm/min (Figure 2). The force leading to fracture (N) was recorded to measure the flexural strength through the following formula: *M* = 3*Wl*/2*bd*2, where *W* is the applied load (N), *l* is the test span (mm), *b* is the specimen width (mm), and *d* is the specimen thickness (mm) [24].

### 2.5. Statistical Analyses

Statistical analyses were done by IBM SPSS for Windows (version 22.0, IBM Corp., Armonk, NY, USA). Kolmogorov–Smirnov and Shapiro–Wilk tests were used to confirm normal distribution and the homogeneity of variances. Two-way ANOVA was used for the descriptive data (mean and standard deviation (SD)), and one-way ANOVA was used to evaluate the effect of each factor, followed by Tukey's post hoc test and independent samples *t*-test for pairwise comparisons (*α* = 0.05).

## 3. Results

Table 1 displays the mean ± SD of the flexural strength in each group. Based on the result of two-way ANOVA, the effect of zirconia type was significant (*P* ≤ 0.001), while the effect of surface treatment with Al_2_O_3_ was insignificant (*P* = 0.249). Moreover, a significant interaction existed between the surface treatment and zirconia type (*P* = 0.01). Having controlled the effect of surface treatment, one-way ANOVA showed that zirconia type significantly affected the flexural strength (*P* ≤ 0.001). Tukey's post hoc test showed that, in both the control and APA subgroups, the flexural strength of both HT and LT zirconia specimens was significantly higher than that in the ML system (*P* ≤ 0.001) (Table 2).

Pairwise comparisons revealed the flexural strength was not significantly different between the LT (*P* = 0.978) and HT (*P* = 0.182) groups in neither the control nor the APA subgroups. Independent *t*-test showed that the surface treatment had no significant effect on the flexural strength in the HT (*P* = 0.176) or LT zirconia group (*P* = 0.110). But, in the ML zirconia system, air-abrasion significantly reduced the flexural strength compared with the control group (*P* = 0.002) (Table 2).

**Figure 3:**
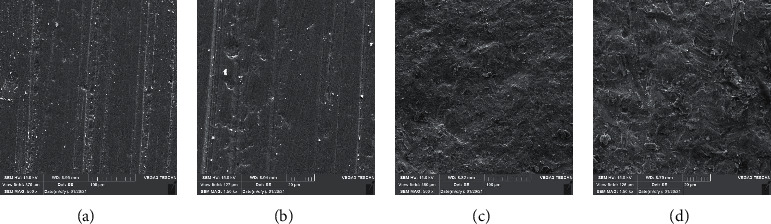
Multilayered zirconia SEM micrographs: (a, b) without APA and (c, d) with APA (×500 and ×1500 magnifications).

**Figure 4:**
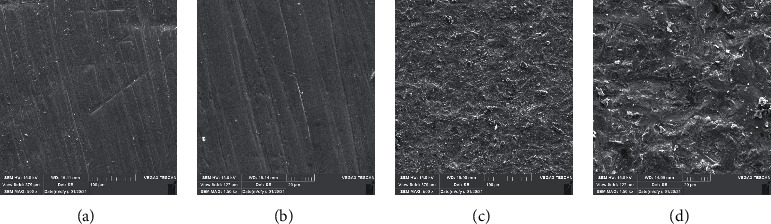
Low-translucent zirconia SEM micrographs: (a, b) without APA and (c, d) with APA (×500 and ×1500 magnifications).

**Figure 5:**
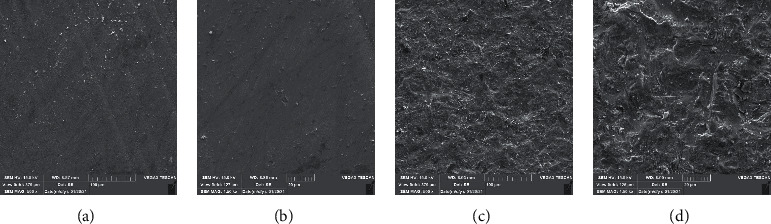
High-translucent zirconia SEM micrograph: (a, b) without APA and (c, d) with APA (×500 and ×1500 magnifications).

SEM images showed that surface treatment with Al_2_O_3_ created porous and irregular surfaces in all types of zirconia. In the control group without surface treatment, ML system and HT zirconia had the most and least irregular surfaces, respectively. However, after airborne abrasion with Al_2_O_3_, LT and HT zirconia showed rougher surfaces (Figure 3–5).

## 4. Discussion

The present findings rejected the null hypothesis since different zirconia translucencies and surface treatment with Al_2_O_3_ considerably influenced the flexural strength of monolithic zirconia. Accordingly, the flexural strength of ML zirconia system was lower than that of LT and HT zirconia, which was in line with some other studies [25, 26].

Most recently, multilayered zirconia systems have been developed to further improve the esthetic properties of dental restorations through mimicking the shade gradient of natural teeth. These systems grow in intensity and opacity towards the gingival region where the incisal area of a crown is most translucent. Completely different grades of such zirconia systems with distinctive properties are advocated for numerous indirect dental restorative applications. The first marketed multilayered zirconia system was Katana (Kuraray Noritake, Japan), which included three grades as ultratranslucent multilayered zirconia, supertranslucent multilayered zirconia, and multilayered zirconia [27]. Flinn et al. [5] examined four translucent Y-TZP materials including Katana ML, Katana HT13, Prettau, and BruxZir. They observed that low thermal degradation of Y-TZP significantly decreased the flexural strength of Prettau and BruxZir; in contrast to the present findings about multilayered zirconia system, Katana ML and Katana HT13 did not have significantly lower flexural strength.

Pereira et al. [26] and Park et al. [25] stated that the flexural strength of multilayered zirconia was mainly affected by the grain size. Presence of common particles with a size slightly larger than the wavelength of incident light could result in different translucencies due to the high mismatch of the index of refraction between the zirconia particles and the matrix [28, 29]. Nanoparticles such as alumina in many traditional ceramic materials account for the fascinating optical properties. Zirconia grain size is determined by factors such as dopants, sintering pressure, temperature, and times. However, the grains <0.2 *μ*m are not usable in smaller dimensions due to the impossibility of phase transition [28]. Spyropoulou et al. [30] evaluated three different shades (light, medium, and intense) and concluded that shaded zirconia was partially translucent.

The current findings showed that the flexural strength was not significantly different between the LT and HT zirconia. Likewise, Matsuzaki et al. [31] found that the strength of translucent tetragonal zirconia polycrystalline (TZP) and opaque TZP was comparable. They also noted that translucent zirconia with different colors could improve the translucency compared with the conventional opaque zirconia.

In line with the present findings about ML zirconia compared with HT and LT zirconia, Mao et al. [32] found that ultratranslucent zirconia (5Y-PZS) was significantly more translucent than the conventional high-cubic containing 3Y-TZP, although it was weaker.

Another finding of the present study was the significant interaction between the surface treatment and zirconia type. APA creates the clean and rough surface required for higher adhesion to dental cement or veneering porcelains [19, 22, 33, 34]. While polishing considerably improves the strength, other surface treatments, namely, coarse grinding and mechanical fatigue do the opposite [35]. Hence, the current study opted to assess the impact of air-abrasion with 110 *μ*m Al_2_O_3_ particles on the ﬂexural strength of monolithic zirconia [22, 23, 34]. With respect to the microscopic morphological evaluation, surface alteration enhanced the surface roughness, more considerably in LT and HT monolithic zirconia. Although their difference was statistically insignificant, APA efficiently improved the flexural strength in both APA subgroups, compared with the control subgroups. However, it was totally different in multilayered zirconia system, as the air-abraded subgroup had lower flexural strength than the control counterpart.

Contrary to what the present study found about LT and HT zirconia, some studies showed concerns about the superficial damage after airborne particle abrasion with 50–120 µm Al_2_O_3_ particles (0.28–0.40 MPa) [36–38]. However, similar to the current findings about LT and HT zirconia, abrasion was found to improve the strength of Y-TZP [19, 39]. Disregarding the aging process, abrasion triggers transformation toughening and forms a resistant protective layer on the surface and consequently improves the strength [39]. Sandblasting duration or distance was not stated as an effective factor [6, 39, 40].

Mao et al. [32] examined the interaction between the surface treatments and flexural strength of ultratranslucent zirconia (5Y-PSZ) in comparison with the conventional high cubic-containing 3Y-TZP. Similar to what the present study found for ML zirconia system, the strength decreased significantly in 5Y-PSZ after high polishing. They stated that despite the increased translucency, high cubic content reduced the strength of zirconia due to decreased tetragonal to monolithic transformation. Sulaiman et al. [9] found that airborne particle abrasion lowered the flexural strength of fully stabilized zirconia, while enhancing the flexural strength of partially stabilized monolithic zirconia. However, they documented that artificial aging affected the flexural strength of neither group. Similar findings were reported by Stawarczyk et al.'s study [41].

A systematic review detected that the translucency and flexural strength were significantly influenced by the composition, microstructure, and surface treatment [35]. Reviewing the effect of sintering temperature on microstructure, flexural strength of a fully stabilized monolithic zirconia, Cardoso et al. [42] noted that higher sintering temperatures increased the grain size but did not change the crystal phase concentration. They also found that different sintering temperatures significantly affected the reflectance and sum of light absorption scattering; nonetheless, it did not significantly influence the translucency parameter, opacity, or flexural strength. Furthermore, Juntavee et al. [43] asserted that flexural strength of translucent monolithic Y-TZP was affected by the alteration of the sintering process, either the sintering temperature or sintered-holding time.

Kurtulmus et al. [44] investigated the influence of surface treating in pre- and postsintering stages on the flexural strength and optical properties of zirconia. They observed that surface treatments in the postsintering stage favorably affected the flexural strength, while presintering surface treatments increased the translucency. In addition, Yilmaz et al. [45] recommended avoiding presintering air-abrasion of zirconia clinically since it decreases the flexural strength.

Among the limitations of the present study, was the nonclinical in vitro nature, which restricts free interpretation of the results for the clinical conditions. Further studies are recommended to consider other types of zirconia with different surface treatments.

## 5. Conclusions

Within the limitations of this study and considering the in vitro conditions, it can be concluded that, between the three different translucencies of monolithic zirconia, the multilayer zirconia system had the lowest flexural strength than the two other types of monolithic zirconia. Besides, the flexural strength of this system decreased by surface treatment with airborne particle abrasion with Al_2_O_3_.

## Figures and Tables

**Table 1:** Mean ± standard deviation of flexural strength of the three zirconia types (MPa) (*P* ≤ 0.05).

Zirconia type	Low translucent	High translucent	Multilayered system
Surface treatment			
No surface treatment	884.6 ± 215.2 ^a A^	863.2 ± 257.1 ^a A^	273.5 ± 19.6 ^b A^
Airborne particle abrasion	1110.7 ± 85.9 ^a A^	1013.2 ± 66 ^a A^	219.3 ± 17 ^b B^

Different lowercase letters indicate differences between the types of zirconia in each surface treatment (row). Different uppercase letters indicate differences between the surface treatment methods in each zirconia system (column).

**Table 2:** The effect of the studied factors on the flexural strength (two-way ANOVA) (*P* < 0.05).

Variables	d*f*	Mean square	F	Sig.
Zirconia type	2	6.131	207.723	0.000
Airborne particle abrasion	1	0.041	1.399	0.249
Zirconia × airborne particle abrasion	2	0.166	5.639	0.010

## Data Availability

The data supporting the findings of this study are available upon request from the corresponding author.
